# N-acetyl-L-cysteine Prevents Lactate-Mediated PGC1-alpha Expression in C2C12 Myotubes

**DOI:** 10.3390/biology8020044

**Published:** 2019-06-10

**Authors:** Minas Nalbandian, Zsolt Radak, Masaki Takeda

**Affiliations:** 1 Graduate School of Medicine, Kyoto University, Kyoto 606-8315, Japan; 2 Research Institute of Sport Science, University of Physical Education, 1525 Budapest, Hungary; radak@tf.hu; 3 Graduate School of Sports and Health Science, Doshisha University, Kyotanabe 610-0394, Japan; mtakeda@mail.doshisha.ac.jp

**Keywords:** lactate signaling, ROS, PGC1-alpha, myotubes

## Abstract

Background: Exercise induces many physiological adaptations. Recently, it has been proposed that some of these adaptations are induced by exercise-mediated lactate production. In this study, we aimed to investigate in vitro the effect of lactate in cultured myotubes and whether antioxidants could inhibit the effect. Methods: Differentiated myotubes were cultured at different concentrations of L-lactate (0, 10, 30, 50 mM) in the absence or presence of an antioxidant, N-acetyl-L-cysteine (Nac). The temporal effect of lactate exposure in myotubes was also explored. Results: Two hours of exposure to 50 mM L-lactate and six hours of exposure to 30 or 50 mM L-lactate caused a significant increase in PGC1-alpha (peroxisome proliferator-activated receptor γ coactivator-1α) expression in the myotubes. This up-regulation was suppressed by 2 mM Nac. Intermittent and continuous lactate exposure caused similar PGC1-alpha up-regulation. These results suggest that the increase in PGC1-alpha expression is mediated by reactive oxygen species (ROS) production from lactate metabolism and that both continuous and intermittent exposure to L-lactate can cause the up-regulation.

## 1. Introduction

Exercise is known to induce physiological adaptations, especially in mitochondria, and many underlying mechanisms and signaling molecules have been identified, including lactate [1,2,3,4]. PGC1-alpha (peroxisome proliferator-activated receptor γ coactivator-1α) is the master regulator of mitochondrial biogenesis [5,6]. Furthermore, it is up-regulated following exercise and is dependent on the exercise intensity [7]. Egan et al. showed that exercise at 80% VO2max (maximum oxygen consumption) up-regulated PGC1-alpha expression to a greater extent compared to the exercise at 40% VO2max [7].

Lactate is known to increase PGC1-alpha expression. In L6 myoblasts cultured with 20 mM of sodium lactate for six hours, PGC1-alpha expression was increased around 30% [8]. In the same study, it was shown that 20 mM of sodium lactate increased reactive oxygen species (ROS) activity. Other study showed that in mice, 1 g/kg of sodium lactate by intraperitoneal injection augmented the PGC1-alpha expression in gastrocnemius 3 hours after lactate injection [9]. Accordingly, it has been proposed that lactate may be one of the molecules that regulate the exercise-mediated PGC1-alpha up-regulation [1,10].

Several studies have examined the effect of exercise intensity on the metabolic adaptations [11,12]. Different exercise intensities have different metabolic demands, which will lead to different adaptations. For example, low intensity-high duration and high intensity-low duration exercise have been studied [7,13,14,15,16], and both of these exercises modalities increase lactate production [15], but whether the increased lactate causes different adaptations is unknown. 

The aim of this research was to study the in vitro effect of lactate exposure on PGC1-alpha expression in cultured myotubes. Based on lactate’s ability to up-regulate PGC1-alpha expression [8,9,10], lactate mediated ROS production activation [7], and the role of ROS in mitochondrial adaptations [10], we hypothesized that the lactate effect is mediated by ROS. Therefore, we tested different concentrations of L-lactate (0, 10, 30, 50 mM) with a widely-used antioxidant, N-acetyl-L-cysteine (Nac). Finally, we studied the effect of intermittent and continuous lactate exposure.

## 2. Materials and Methods

### 2.1. Cell Culture

C2C12 myoblasts were cultured in a 6-well plate (20,000 cells/cm^2^). Briefly, cells were cultured in proliferation medium (Dulbecco’s modified Eagle’s medium (DMEM) containing 10% fetal bovine serum). After 80–90% confluence was reached, the cells were cultured in differentiation medium (DM; DMEM containing 2% horse serum). Two days after differentiation, the cells were cultured for two or six hours in DM with sodium L-lactate (Sigma Aldrich, Co., St. Louis, MO, USA) at different concentrations (0, 10, 30, 50 mM) and patterns (intermittent or continuous). To determine the role of reactive oxygen species (ROS) on downstream lactate-mediated signaling, in some experiments, cells were cultured with 2 mM Nac. Immediately, 2 h, 3 h, or 6 h after the cells were cultured with lactate, total RNA or proteins were obtained for further analysis. All the experiments were performed three independent times, and three replicates were used each time.

### 2.2. Real-Time RT-PCR

Total RNA was extracted using ISOGEN II (Nippon Gene, Tokyo, Japan). After total RNA isolation, cDNA was synthesized using a PrimScript TM II first strand cDNA Synthesis Kit (TKR, Shiga, Japan) following the manufacturer’s protocol. For real-time RT-PCR, RNA was reverse-transcribed using KAPA SYBR FAST qPCR Kit Master Mix ABI PrismTM (KAPA BIO, Wilmington, MA, USA) and later amplified on the Applied Biosystems StepOne Real-Time PCR System (Applied Biosystems, Waltham, MA, USA). The amplification protocol included an initial denaturation step for 10 min at 95 °C followed by 40 cycles of denaturation for 15 s at 95 °C, annealing for 1 min at 60 °C, and extension for 1 min at 72 °C. Relative expressions were normalized to 18S ribosomal RNA using the DDCt method. The amplification of specific transcripts was confirmed by obtaining melting curves between 68–95°C at the end of the PCR. The sequences of the primers used are PGC1-alpha: forward = 5´-CAC CGT AAA TCT GCG GGA TG-3´, reverse = 5´-TAT CCA TTC TCA AGA GCA GCG AAA G-3´; SIRT1: forward = 5´-GCA ACA GCA TCT TGC CTG AT-3´, reverse = 5´GTG CTA CTG CTC TCA CTT-3´; Cyt C (cytochrome c): forward = 5´-ATA GGG GCA TGT CAC AAA C- 3´, reverse = 5´-GTG GTT AGC CAT AAA G-3´; S18: forward = 5´-TTC TGG CCA ACG GTC TAG ACA AC-3´, reverse = 5´-CCA GTG GTC TTG GTG TGC TGA-3´.

### 2.3. Immunoblots

Cells were washed with phosphate-buffered saline (PBS) and homogenized in EzRIPA lysis buffer (20 mM HEPES pH 7.5, 1% NP-40, 0.1% SDS, 0.5% deoxycholic acid, 150 mM sodium chloride), supplemented with protease and phosphatase inhibitors (ATTO, Tokyo, Japan). Later the homogenate was incubated for 15 min on ice and centrifuged (15 min at 14,000 g and 4 °C). The supernatant was obtained and purified by the second round of centrifugation (same conditions). Samples were stored at −80 °C until analyzed. The samples were found to not contain significantly different amounts of total protein; therefore, identical volumes from each sample were mixed with Laemmli sample buffer and heated for 2 min at 95 °C. After separation on 8–12.5% SDS-PAGE gels, proteins were transferred to PVDF membranes (ATTO, Tokyo, Japan), which were blocked for 60 min with TBS containing 0.1% Tween-20 (TBS-T) and 5% skim milk or with Block Ace Powder (DSP, Osaka, Japan) dissolved in purified water. Membranes were then probed at 4 °C overnight in TBS-T containing 0.4% NaN3 and 1:1000 dilutions of specific antibodies against PGC1-alpha (Novus Biologicals, Centennial, CO, USA) and GAPDH (Abcam, Cambridge, UK). Subsequently, the membranes were labeled for 60 min with 1:2500 dilutions of anti-rabbit or anti-mouse immunoglobulin G (GE Healthcare, Buckinghamshire, UK). Bands were visualized using the ECL Prime system (GE Healthcare, Buckinghamshire, UK) and quantified on the ChemiDocTM MP system (Bio-Rad, Hercules, CA, USA). Protein abundance was normalized to GAPDH.

### 2.4. Statistics

Data are reported as means ± S.E. and tested for significance using one-way ANOVA with Bonferroni’s post hoc. Differences between groups were considered significant when *p* < 0.05.

## 3. Results

### 3.1. PGC1-alpha Expression Depends on Lactate Concentration and Exposure Time

**Figure 1:**
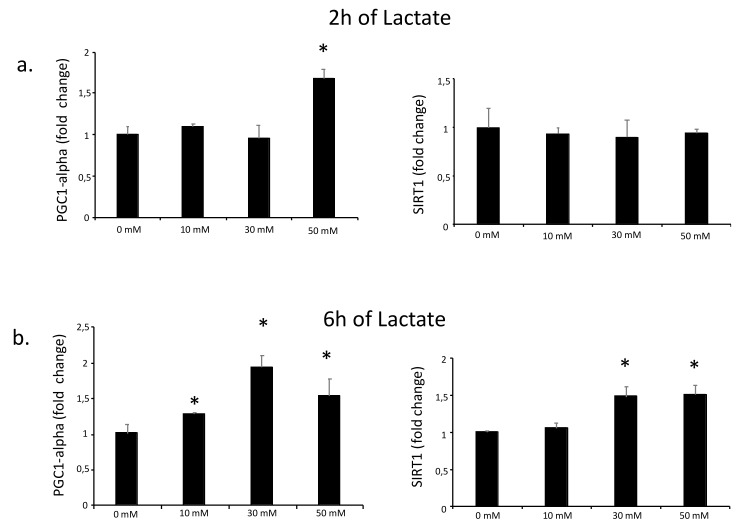
qPCR analysis of PGC1-alpha and SIRT1 expression in C2C12 myotubes cultured with L-lactate. C2C12 myotubes were cultured for 2 (**a**) and 6 h (**b**) with L-lactate at different concentrations (0, 10, 30, and 50 mM). In the time between lactate culture and sample collection (one hour), cells were cultured with DM. Error bars show standard deviations. * *p* < 0.05 from 0 mM. All the experiments were performed three independent times with three replicates each time.

To investigate whether lactate up-regulates PGC1-alpha expression, C2C12 myotubes were cultured with sodium lactate (0, 10, 30, and 50 mM) for 2 or 6 h. After culture with lactate, the cells were kept in DM for one hour and lysed for RNA extraction. Two hours of exposure to 50 mM lactate and six hours of exposure to all three lactate concentrations significantly increased the PGC1-alpha expression (Figure 1). We also measured the expression of the NAD+ (Nicotinamide adenine dinucleotide)-dependent protein deacetylase sirtuin 1 (SIRT1), which acts together with PGC1-alpha to form an energy-sensing network that regulates metabolism [17]. Lactate did not affect SIRT1 expression at 2 h exposure, but 30 and 50 mM lactate did at 6 hours (Figure 1). These results suggest that lactate has the potential to increase PGC1-alpha expression in myotubes. 

### 3.2. Lactate-Mediated Up-regulation of PGC1-alpha Is Blocked by N-acetyl-L-cysteine (Nac)

**Figure 2:**
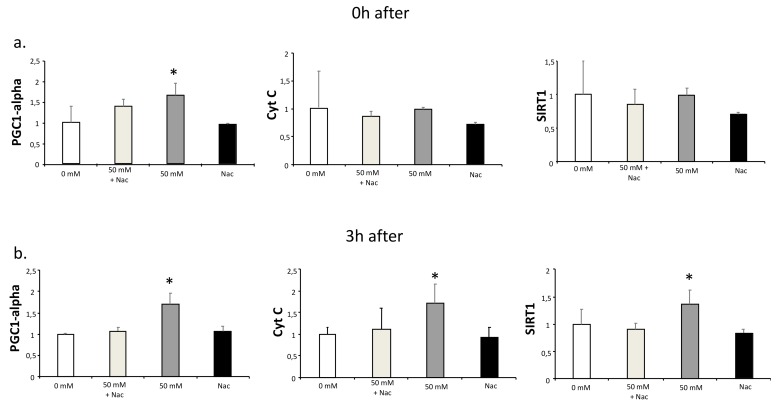
qPCR analysis of PGC1-alpha, Cyt C, and SIRT1 in C2C12 myotubes cultured with lactate and N-acetyl-L-cysteine (Nac). C2C12 myotubes were cultured for 2 h with L-lactate (0 and 50 mM) in the presence or absence of Nac (2 mM). Samples for gene expression analysis were obtained at 0 (**a**) and 3 h (**b**) after the culture with lactate and Nac. In the time between lactate culture and sample collection (0 to 3 h), cells were cultured with DM. Error bars show standard deviations. * *p* < 0.05 from 0 mM. All the experiments were performed three independent times with three replicates each time.

It has been reported that lactate increases ROS production, and this production has been suggested to be responsible for lactate-induced adaptations [1,2,4]. To test this hypothesis, we investigated if antioxidant treatment could inhibit the lactate-induced increased expression of PGC1-alpha. For this purpose, C2C12 myotubes were cultured for two hours with or without 50 mM lactate and with or without 2 mM Nac. RNA samples were obtained immediately after and three hours after the culture. PGC1-alpha expression was significantly increased at both times at 50 mM lactate, as were the expressions of its downstream genes, Cyt C and SIRT1 [18] (Figure 2). Nac inhibited the up-regulation of all three genes (Figure 2). These results indicate that part of the lactate-mediated PGC1-alpha up-regulation may be because of ROS production.

### 3.3. Lactate Intermittency Is an Efficient Way to Increase PGC1-alpha Expression 

Intermittent exercise, which consists of repeated sets of exercise separated by periods of recovery, is suggested to produce comparable physiological adaptations to continuous exercise [13,19]. During intermittent exercise, lactate production also follows an intermittent pattern [20]. Therefore, to examine if intermittent exposure to lactate has the potential to induce mitochondrial adaptations, we cultured C2C12 myotubes for 2 h, but by changing the medium every 15 min (DM supplied with different concentrations of L-lactate; see Table 1).

**Figure 3:**
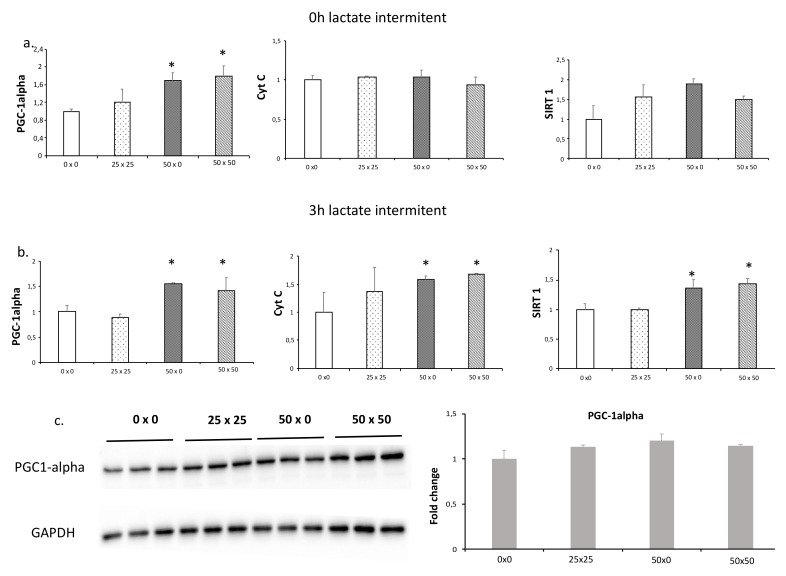
qPCR analysis for intermittent and continuous exposure to lactate in C2C12 myotubes and Western blot of PGC1-alpha after continuous or intermittent exposure to lactate. C2C12 myotubes were cultured with different concentration patterns of L-lactate (see Table 1). Samples for qPCR analysis were obtained 0 (**a**) and 3 h (**b**) after the exposure series. Samples for Western blot (**c**) were obtained 3 h after the exposure series (see Table 1). In the time between lactate culture and sample collection (zero or three hours), cells were cultured with DM. Error bars show standard deviations. * *p* < 0.05 from non-lactate exposure (0 × 0). RNA experiments were performed three independent times with three replicates each time, and protein experiments with three independent samples.

Immediately after the lactate treatment, PGC1-alpha was equally up-regulated at 50 × 0 and 50 × 50 conditions (Figure 3a), indicating that intermittent (50 × 0) and continuous (50 × 50) exposure have similar effects. Cyt C and SIRT1 were only up-regulated 3 hours after in 50 × 0 and 50 × 50 conditions (Figure 3a). It was reported that 30 min was enough to increase PGC1-alpha protein levels (20%) in skeletal muscles after exercise with an increase (40%) until 5 h [21]. In the present study, we evaluated the PGC1-alpha expression and protein levels three hours after the two-hour culture, and we found PGC1-alpha expression was still up-regulated in 50 × 0 and 50 × 50 conditions (Figure 3b). Finally, western blotting showed that PGC1-alpha protein levels 3 hours after lactate exposure were unchanged between intermittent and continuous exposure (Figure 3c).

## 4. Discussion

The main findings of this study are that lactate increases PGC1-alpha expression in C2C12 myotubes in a concentration-dependent manner, and this effect can be in part prevented by Nac, suggesting that one of the mediators of the PGC1-alpha up-regulation is ROS production. 

It was reported that four bouts of 4 min of cycling exercise performed at 85% of VO2 increased PGC1-alpha expression, as well as venous blood lactate concentration, in comparison with the same amount of exercise at 70% of VO2max [22]. Additionally, moderate intensity exercise up-regulates mouse skeletal muscle PGC1-alpha expression to a greater extent compared to low-intensity exercise [23]. In agreement with these studies, we found that a higher concentration of lactate (30 and 50 mM) increased PGC1-alpha in cultured myotubes. Furthermore, Cyt C (a downstream protein of PGC1-alpha) was increased at 3 h after 50 mM lactate exposure.

SIRT 1 has been reported as a downstream target of PGC1-alpha, which regulates fatty acid oxidation genes in mitochondria [6]. We found that 50 mM and 20 mM of lactate could induce SIRT1 expression. Moreover, with 50 mM lactate and Nac exposure, lactate up-regulation of SIRT1 expression was suppressed, suggesting that SIRT1 up-regulation may be mediated by PGC1-alpha lactate-mediated overexpression.

Additionally, it has been reported that intermittent and continuous exercise produces similar acute physiological adaptations [15]. Cochran et al. reported that four 30 s Wingate tests interspersed with 4 min of rest, or a 4 min bout of continuous exercise (matched for total work), produces similar acute increases in markers of AMPK (adenosine monophosphate-activated protein kinase) and p38 mitogen-activated protein kinase activation and PGC1-alpha expression in vastus lateralis [15]. Indeed, with our experimental design, we observed that continuous and intermittent exposure to lactate up-regulated PGC1-alpha similarly.

We also examined the protein levels of PGC1-alpha because it has been reported to be regulated by exercise [24,25,26]. We found a slight but insignificant increase. This finding may indicate that a long time and more than one dose of lactate exposure is needed to significantly induce the protein [16,27]. It should be mentioned that in the present study, we used lactate concentrations (i.e., 50 mM) far above physiological levels; therefore, in vivo studies should be performed to further confirm our results. Moreover, other pathways, such as AMPK pathways, were not studied in this research, but the regulation of NAD/NADH by the lactate metabolism may affect AMPK signaling independent of ROS to cause skeletal muscle adaptations [1]. Additionally, because lactate is a highly transportable molecule [14,28,29,30], it can travel to many tissues, such as the brain, liver, fat, and heart. Further research should be done to understand the role of exercise-produced lactate in tissues other than skeletal muscle. 

## 5. Conclusions

In conclusion, this study showed that lactate could induce PGC1-alpha gene expression in myotubes and that this effect could be diminished by Nac administration. Further studies should confirm these results in vivo and detail other signaling pathways involved in lactate-mediated adaptations. Additionally, tissue types, besides myotubes, should be investigated. 

## Figures and Tables

**Table 1:** Intermittent patterns of lactate exposure.

Pattern Name	Lactate Concentration in Interval 1 (mM)	Lactate Concentration in Interval 2 (mM)
0 × 0	0	0
25 × 25	25	25
50 × 0	50	0
50 × 50	50	50

One cycle consisted of Interval 1 and Interval 2 in that order. One exposure series had four cycles for a total of 2 h. In every condition, the medium was changed every 15 min.
